# Developing a new assessment model for systemic lupus erythematosus disease activity based on a case cohort

**DOI:** 10.3389/fimmu.2026.1742231

**Published:** 2026-03-19

**Authors:** Dan Liu, Ke Ma, Jing-xuan Liu, Da-wei Wen, Meng Han, Yi-heng Xu, Meng-rui Zhang, Hong-da Liang, Yi-Jing Zhang, Ming Li

**Affiliations:** 1 Department of Rheumatology and Immunology, Shandong Provincial Hospital Affiliated to Shandong First Medical University (Shandong Provincial Hospital), Jinan, China; 2 Department of Rheumatology and Immunology, Peking University People's Hospital, Qingdao; Women and Children's Hospital, Qingdao University, Qingdao, China; 3 Department of Rheumatology and Immunology, The Affiliated Hospital of Qingdao University, Qingdao, China; 4 Department of Rheumatology and Immunology, Beijing University of Chinese Medicine East Hospital, Zaozhuang Hospital, Zaozhuang, China; 5 Department of Gastroenterology in Health Building, Shandong Provincial Hospital Affiliated to Shandong First Medical University (Shandong Provincial Hospital), Jinan, China

**Keywords:** assessment model, PGA, SLE activity score, SLEDAI-2K, systemic lupus erythematosus

## Abstract

**Objective:**

To develop and validate a novel SLE disease activity scoring model designed to enhance diagnostic capability for moderate-to-severe disease activity in SLE patients compared to the SLE Disease Activity Index 2000 (SLEDAI-2K).

**Methods:**

All 1163 SLE patients from Shandong Provincial Hospital and the Affiliated Hospital of Qingdao University constituted the derivation cohort, while another 323 patients from Shandong Provincial Hospital served as the validation cohort. Disease activity was assessed for each patient using the Physician Global Assessment (PGA) and SLEDAI-2K. With PGA-defined moderate-to-severe SLE activity as the dependent variable, binary logistic regression analysis identified factors influencing disease activity and constructed a regression model. Receiver operating characteristic (ROC) curve analysis evaluated the model’s discriminative ability. Correlations between the new scoring model, PGA, and SLEDAI-2K were examined.

**Results:**

Binary logistic regression identified 25 clinical manifestations as independent risk factors for higher SLE activity (all P<0.05): neuropsychiatric symptoms, visual impairment, vasculitis, arthritis, myositis, hematuria, proteinuria, pyuria, alopecia, rash, mucosal ulcers, pleurisy, pericarditis, hypocomplementemia, elevated anti-dsDNA, fever, thrombocytopenia, leukopenia, pulmonary hypertension, hypothyroidism, hypocalcemia, lymphadenopathy, abnormal liver function, high-titer ANUA. Using the 25 variables listed above, we constructed a new scoring model. ROC analysis for distinguishing moderate-to-severe activity showed areas under the curve of 0.972 (95% CI: 0.963-0.980, P<0.001) in the derivation cohort and 0.971 (95% CI: 0.958–0.985, P<0.001) in the validation cohort.

**Conclusion:**

Twenty-five clinical manifestations were identified as independent risk factors for assessing moderate-to-severe SLE activity. The resulting model demonstrated enhanced accuracy in identifying moderate-to-severe disease activity states.

## 1 Introduction

SLE is a complex autoimmune disease characterized by multisystem involvement, significant variability in severity, an unpredictable disease course (often manifesting as alternating periods of remission and relapse), and highly heterogeneous disease progression (1). Without timely intervention, it can lead to severe damage to multiple organs and a significantly increased risk of infections. Given that SLE disease activity is a key predictor of organ damage and mortality (2), its timely and accurate quantitative assessment is crucial.

Medical scoring systems play a central role in assessing SLE disease activity. By integrating multisystem clinical manifestations and laboratory parameters from patients, these systems categorize disease activity states as mild, moderate, or severe. This stratification guides clinicians in developing more individualized treatment plans and holds significant value in preventing SLE-related complications (3). Commonly used clinical assessment tools include the Systemic Lupus Erythematosus Disease Activity Index (SLEDAI), its updated version SLEDAI-2K, the Systemic Lupus Activity Measure (SLAM), the British Isles Lupus Assessment Group Index-2004 (BILAG-2004), the European Consensus Lupus Activity Measurement (ECLAM), the SLE Responder Index (SRI), and the emerging SLE Disease Activity Score (SLE-DAS) (4). These methods differ in their assessment dimensions and weighting of components (5). However, in practice, due to the broad spectrum of SLE involvement and the diverse clinical presentations among patients, these scoring systems all possess significant limitations (5).

Currently, the most frequently used SLE activity scoring method in clinical practice is SLEDAI-2K. SLEDAI-2K incorporates 24 clinical and laboratory indicators, covering manifestations across multiple systems including mucocutaneous (e.g., rash, alopecia), musculoskeletal (arthritis), renal (e.g., proteinuria, hematuria), hematological (thrombocytopenia), immunological (positive anti-dsDNA antibodies, low complement), and neurological (seizures, psychosis) (6).

However, the SLEDAI-2K scoring system has several limitations. First, its assessment scope is strictly confined to active inflammatory manifestations that are new or significantly worsened within the 10 days prior to evaluation. It fails to incorporate the impact of chronic, irreversible cumulative organ damage (e.g., joint deformity, renal fibrosis) on the patient’s overall disease state and prognosis. Second, its reliance on a fixed 10-day time window makes it difficult to effectively capture and reflect symptoms with intermittent or fluctuating characteristics (such as periodic fever, fatigue). Most critically, SLEDAI-2K exhibits significant deficiencies in comprehensively assessing the activity of multisystem involvement in SLE. The score fails to adequately incorporate clinical manifestations and laboratory parameters related to key organ systems, including the digestive system (e.g., abnormal liver function, pancreatitis), circulatory system (e.g., pericarditis, myocarditis, pulmonary hypertension), and respiratory system (e.g., pleuritis, pneumonitis). This limitation hinders SLEDAI-2K’s ability to accurately reflect SLE activity.

Unlike the scoring methods mentioned above, in the 2019 updated EULAR management recommendations for SLE, the Physician Global Assessment (PGA) score is the only evaluation tool formally incorporated into the recommendations (7). The core value of PGA lies in its reliance on the clinician’s comprehensive judgment of the patient’s overall condition to quantify the current degree of disease activity (8). Its strengths include the ability to rapidly reflect disease trends during routine clinical encounters and its capacity to integrate all active clinical manifestations and key laboratory parameters, thereby capturing features missed by tools like SLEDAI-2K. Studies confirm that PGA correlates significantly with treatment adjustments and progression of organ damage, serves as a sensitive indicator for assessing treatment effectiveness, and plays an important role in aiding therapeutic decision-making (9).

Considering the limitations of existing standardized scoring tools and the important status of PGA as a guideline-recommended instrument, we are committed to developing and validating a novel SLE activity scoring tool. This tool aims to achieve maximum alignment with PGA scores while addressing the shortcomings of previous scoring methods, particularly by expanding coverage to encompass more affected systems (such as digestive, circulatory, and respiratory systems). Ultimately, our goal is to establish a new standard capable of providing a more accurate and comprehensive assessment of SLE disease activity, thereby offering a stronger foundation for optimizing clinical decision-making.

## 2 Patients and methods

### 2.1 Patients

A total of 1163 hospitalized SLE patients were selected from Shandong Provincial Hospital and the Affiliated Hospital of Qingdao University between 2022 and 2023, including 117 males and 1046 females. Inclusion criteria: Patients fulfilled the revised SLE classification criteria established by the American College of Rheumatology (ACR 2009) (10).

### 2.2 Methods

#### 2.2.1 Data collection

During patient visits, comprehensive clinical data were collected, encompassing all clinical manifestations included in SLEDAI-2K. Additionally, signs frequently observed in SLE patients but not specified in SLEDAI-2K, along with abnormal laboratory results, were systematically documented. Signs included: pulmonary hypertension, thyroid nodules, lymphadenopathy, interstitial pneumonia, splenomegaly, and venous thrombosis. Laboratory results included: albumin, transaminases (AST/ALT) and gamma-glutamyl transferase (GGT), creatinine, blood urea nitrogen (BUN), hemoglobin, serum calcium, serum potassium, serum sodium, C-reactive protein (CRP), erythrocyte sedimentation rate (ESR), immunoglobulin G (IgG), immunoglobulin A (IgA), immunoglobulin M (IgM), antinuclear antibodies (ANA), anti-nucleosome antibodies (AnuA), anti-ribosomal P protein antibodies (anti-rRNP), anti-ribonucleoprotein U1 (anti-U1RNP), anti-centromere antibodies (ACA), anti-histone antibodies (AHA), anti-cardiolipin antibodies (ACL-IgG, ACL-IgM, ACL-IgA), anti-Smith antibodies (anti-Sm), anti-Sjögren’s-syndrome-related antigen A antibodies (anti-SSA), anti-Sjögren’s-syndrome-related antigen B antibodies (anti-SSB), anti-topoisomerase I antibodies (anti-Scl-70), and anti-histidyl-tRNA synthetase antibodies (anti-Jo-1). Concurrently, the SLEDAI-2K score was calculated and the Physician Global Assessment (PGA) score was recorded for each patient. Patients were stratified into SLE disease activity groups—mild activity or moderate-to-severe activity—based on PGA-defined severity levels.

All clinical symptoms were assessed using a time window consistent with SLEDAI-2K, meaning that only manifestations that newly appeared or significantly worsened within the 10 days prior to the visit were recorded (11).

#### 2.2.2 Data processing

Statistical analysis was performed using SPSS software (version 27.0). A P-value < 0.05 was considered statistically significant. The Shapiro-Wilk test was used to assess the normality of continuous variables. Normally distributed continuous variables were expressed as mean ± standard deviation (SD); non-normally distributed continuous variables were expressed as median and interquartile range (IQR). Categorical variables were presented as frequencies and percentages.

#### 2.2.3 Statistical methods

Univariate binary logistic regression analysis was performed on clinical data between the mild SLE activity group and the moderate-to-severe SLE activity group. Variables with P < 0.05 in the univariate analysis underwent collinearity diagnostics; variables with a variance inflation factor (VIF) > 5 were excluded. The retained variables were then entered into a multivariate binary logistic regression analysis using the forward stepwise likelihood ratio method (Forward: LR), ultimately establishing a multivariate logistic predictive model for moderate-to-severe SLE activity. The Hosmer-Lemeshow goodness-of-fit test and Receiver Operating Characteristic (ROC) curve analysis were performed. A simplified scoring system for SLE activity assessment was constructed based on significant variables from the predictive model. The Hosmer-Lemeshow goodness-of-fit test and ROC curve analysis were applied to this scoring system. The maximum Youden index was calculated to determine the optimal diagnostic cutoff value for the score. The area under the curve (AUC) of the ROC curve for the new scoring model was compared with that of the SLEDAI-2K score. Correlation analysis was used to examine the relationships between the new scoring model, the SLEDAI-2K score, and the PGA score. The new scoring model was applied to the validation cohort of 323 patients. Moderate-to-severe SLE activity in the validation cohort was defined as PGA > 1. ROC curve analysis was used to validate the diagnostic sensitivity and specificity of the new scoring model for moderate-to-severe SLE activity and to compare its diagnostic performance with that of SLEDAI-2K.

#### 2.2.4 Ethic approval and consent to participate

The study was conducted in accordance with the principles of the Declaration of Helsinki. This study was approved by the Ethics Committee of Shandong Provincial Hospital Affiliated to Shandong First Medical University (SWYX: NO.2025-118), and the need for informed consent was waived as none of the patients were at risk.

#### 2.2.5 Data availability

The data that support the findings of this study are available from Shandong Provincial Hospital Affiliated to Shandong First Medical University but restrictions apply to the availability of these data, and so are not publicly available. Data are however available from the authors upon reasonable request and with permission of Shandong Provincial Hospital Affiliated to Shandong First Medical University.

## 3 Results

### 3.1 Baseline characteristics of enrolled patients

A total of 1163 SLE patients were included in the model derivation phase, and 323 SLE patients were included in the model validation phase; the baseline characteristics of the enrolled patients are presented in **Table 1**.

**Table 1:** Baseline characteristics of included patients.

Characteristic	Derivation cohort (n=1163)	Validation cohort (n=323)
Age, mean ± SD (years)	38.78 ± 15.16	38.31 ± 13.29
Female gender, n (%)	1046 (89.9%)	284 (87.9%)
course of disease, mean ± SD (months)	54.03 ± 69.39	65.57 ± 65.54
PGA, median (range)	1.64 ± 0.87	1.16 ± 0.58
SLEDAI-2K, median (range)	12.81 ± 7.83	11.66 ± 6.21

### 3.2 Univariate binary logistic regression analysis

To identify potential risk factors for moderate-to-severe disease activity in SLE patients, patient-related medical history and common laboratory indicators not included in SLEDAI-2K were individually incorporated into a univariate binary logistic regression analysis, with statistical significance defined as P < 0.05 (**Table 2**). The analysis revealed that ESR, CRP, IgG, BUN, high-titer ANA, high-titer anti-Sm, high-titer anti-U1RNP, high-titer AHA, AnuA positivity, pulmonary hypertension, hypothyroidism, hypocalcemia, lymphadenopathy, and abnormal liver function (Elevation of any one of ALT, AST, GGT above normal range)were risk factors for moderate-to-severe SLE disease activity, whereas albumin (ALB) and hemoglobin concentration served as protective factors.

**Table 2:** Univariate binary logistic regression analysis of moderate-to-severe disease activity in SLE patients.

Variables	OR (95%CI)	P
ESR	1.009 (1.005-1.013)	<0.001
CRP	1.007 (1.000-1.014)	0.038
IgG	1.023(1.007-1.040)	0.005
BUN	1.136(1.081-1.193)	<0.001
ALB	0.930(0.916-0.945)	<0.001
Hemoglobin concentration	0.980 (0.975-0.986)	<0.001
ANA(3)	4.625(1.335-16.026)	0.016
SM(3)	1.987(1.301-3.305)	0.001
U1RNP (3)	1.724(1.267-2.345)	0.001
ANUA (1)	1.961(1.325-2.902)	0.001
ANUA (2)	1.794(1.217-2.644)	0.003
ANUA (3)	6.792(4.174-11.052)	<0.001
AHA(3)	2.689(1.591-4.546)	<0.001
pulmonary hypertension	3.842 (1.973-7.480)	<0.001
lymphadenopathy	3.137(22.70-4.335)	<0.001
Splenomegaly	2.344(1.308-4.200)	0.004
abnormal liver function	5.036(3.330-7.617)	<0.001
Hyponatremia	5.076(3.044-10.694)	<0.001
Hyperkalemia	2.648(1.670-4.201)	<0.001
Hypocalcemia	10.119(5.440-18.824)	<0.001
hypothyroidism	4.176(2.077-8.394)	<0.001

The numbers after autoantibodies such as ANA, U1RNP, ANUA, AHA, SM, etc. are the level of antibody titer, (1) is low titer; (2) is medium titer; (3) is high titer.

### 3.3 Multivariate binary logistic regression

Collinearity diagnostics were performed on all variables that had shown statistical significance in the univariate binary logistic regression and on all items contained in the SLEDAI-2K. Variables with a variance inflation factor (VIF) ≥ 5 were excluded to remove substantial collinearity. After this exclusion, the remaining indicators were entered into a multivariate binary logistic regression with the forward stepwise likelihood-ratio (Forward: LR) method.

Before running the multivariate analysis, the data were further processed. Because the positive counts of several SLEDAI-2K items—seizures, psychosis, neuropathy, cranial neuropathy, lupus headache and cerebrovascular disease—were low (< 10 patients with ≥ 2 concurrent manifestations), these were collapsed into a single “neuropsychiatric symptoms” category to avoid extreme values and to facilitate subsequent clinical use of the scoring model.

Continuous variables (ESR, CRP, IgG, ALB, BUN, hemoglobin concentration) exhibited significant coefficient discrepancies when included alongside binary variables in multivariate binary logistic regression. To mitigate this and enhance scoring model utility, these variables were dichotomized using cutoff values determined by maximum Youden index: ESR = 13.05 mm/h, CRP = 0.795 mg/L, IgG=19.55g/L, ALB = 33.95g/L, BUN = 5.64mmol/L, hemoglobin concentration=114.5 g/L. Based on clinical relevance for SLE activity scoring, the six variables were transformed as follows: ESR >13.05 mm/h, CRP >0.795 mg/L, IgG >19.55 g/L, ALB <33.95 g/L, BUN >5.64 mmol/L, hemoglobin concentration <114.5 g/L.

**Figure 1:**
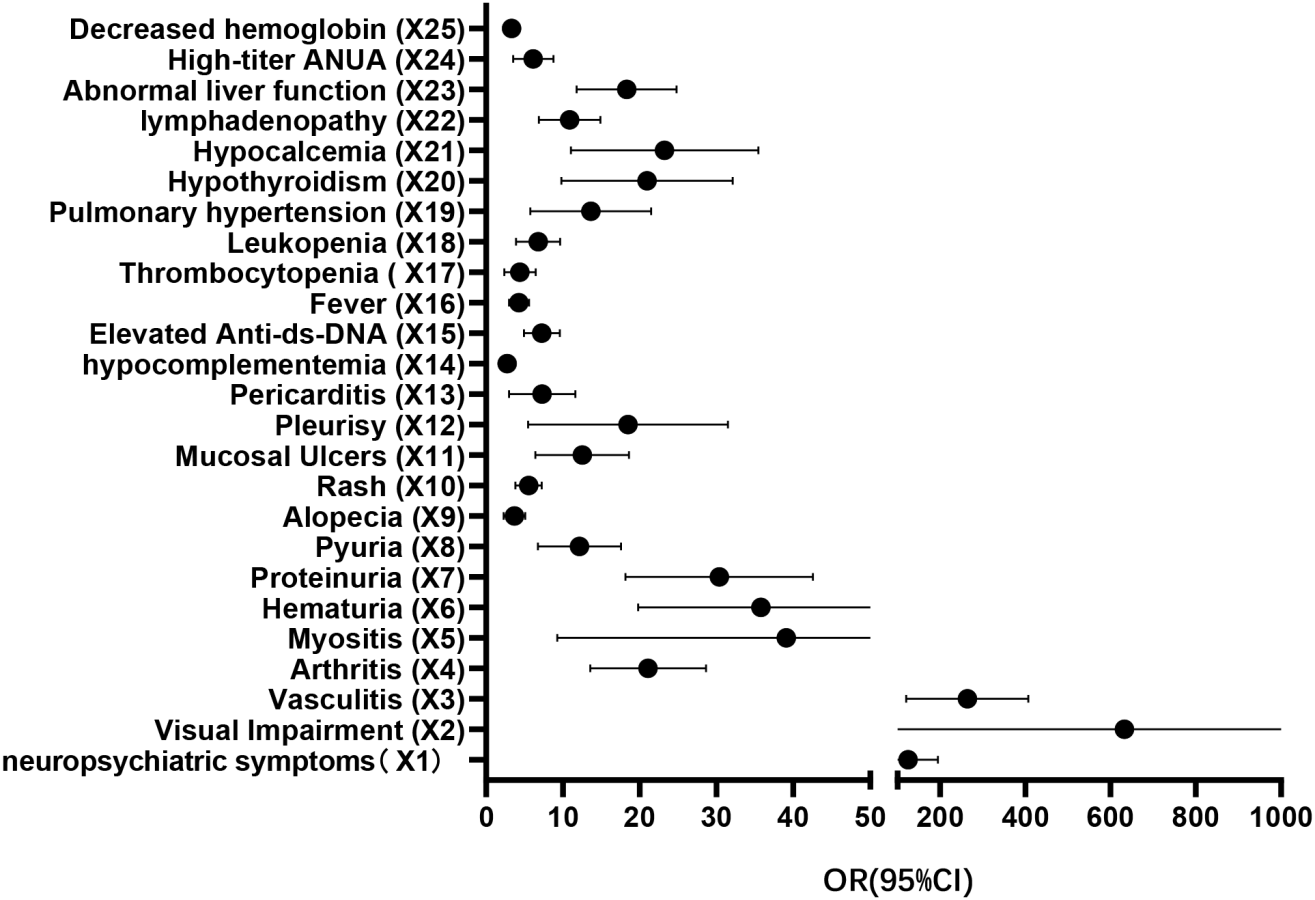
Multivariate binary logistic regression.

This study ultimately identifying 25 factors significantly associated with moderate-to-severe SLE disease activity (**Table 3**) (**Figure 1**). Neuropsychiatric symptoms, visual impairment, vasculitis, arthritis, myositis, hematuria, proteinuria, pyuria, alopecia, rash, mucosal ulcers, pleuritis, pericarditis, hypocomplementemia, elevated anti-dsDNA, fever, thrombocytopenia, leukopenia, pulmonary hypertension, hypothyroidism, hypocalcemia, lymphadenopathy, abnormal liver function, high-titer ANUA, and decreased hemoglobin (hemoglobin concentration less than 114.5 g/l) were independent risk factors for moderate to severe disease activity in patients with SLE.

**Table 3:** Multivariate binary logistic regression analysis of moderate-to-severe disease activity in SLE patients.

Variables	β	OR (95%CI)	P
neuropsychiatric symptoms (X1)	4.438	84.584 (27.323-261.847)	0.000
Visual Impairment (X2)	4.582	97.723 (5.319-1795.401)	0.002
Vasculitis (X3)	5.227	186.299 (64.150-541.034)	0.000
Arthritis (X4)	2.912	18.396 (9.597-35.264)	0.000
Myositis (X5)	2.794	16.345 (2.721-98.182)	0.002
Hematuria (X6)	3.355	28.654 (12.376-66.343)	0.000
Proteinuria (X7)	3.236	25.442 (12.092-53.531)	0.000
Pyuria (X8)	2.278	9.760 (4.240-22.462)	0.000
Alopecia (X9)	1.153	3.168 (1.576-6.365)	0.001
Rash (X10)	1.607	4.990 (2.840-8.769)	0.000
Mucosal Ulcers (X11)	2.258	9.561 (3.778-24.199)	0.000
Pleurisy (X12)	2.233	9.326 (1.971-44.135)	0.005
Pericarditis (X13)	1.556	4.739 (1.425-15.760)	0.011
hypocomplementemia (X14)	0.921	2.513 (1.503-4.200)	0.000
Elevated Anti-ds-DNA (X15)	1.875	6.522 (3.665-11.606)	0.000
Fever (X16)	1.370	3.934 (2.221-6.967)	0.000
Thrombocytopenia (X17)	1.247	3.478 (1.455-8.312)	0.005
Leukopenia (X18)	1.718	5.571 (2.541-12.215)	0.000
Pulmonary hypertension (X19)	2.207	9.085 (2.852-28.942)	0.000
Hypothyroidism (X20)	2.711	15.038 (5.315-42.547)	0.000
Hypocalcemia (X21)	2.823	16.826 (6.046-46.829)	0.000
lymphadenopathy (X22)	2.241	9.403 (4.798-18.426)	0.000
Abnormal liver function (X23)	2.771	15.980 (8.364-30.531)	0.000
High-titer ANUA (X24)	1.615	5.029 (2.271-11.135)	0.000
Decreased hemoglobin (X25)	1.110	3.036 (1.806-5.103)	0.000
constant	-8.675	0.000 (—)	0.000

A predictive model for severe activity in the disease of SLE patients was developed based on the variables derived from multifactorial binary logistic regression analysis, which resulted in the model: Logit(P)=-8.675 + 4.438X1 + 4.582X2 + 5.227X3 + 2.912X4 + 2.794X5 + 3.355X6 + 3.236X7 + 2.278X8 + 1.153X9 + 1.607X10 + 2.258X11 + 2.233X12 + 1.556X13 + 0.921X14 + 1.875X15 + 1.370X16 + 1.247X17 + 1.718X18 + 2.207X19 + 2.711X20 + 2.823X21 + 2.241X22 + 2.771X23 + 1.615X24 + 1.110X25. Hosmer-Lemeshow goodness-of-fit test and ROC curve analysis were done for the prediction model. The Hosmer-Lemeshow goodness-of-fit test χ² = 6.173 was calculated with P = 0.628 > 0.05, indicating that the predictive model is well calibrated. The ROC curve was plotted according to the predicted value of its occurrence probability, and the AUC of the ROC curve was 0.972 (95% CI: 0.963-0.980, P < 0.001), which indicated that the differentiation of the prediction model was good. The maximum Yoden index was calculated to be 0.829, with a sensitivity of 92.7% and a specificity of 90.2%.

### 3.4 Construction of disease activity scoring system for SLE patients

It is proposed to construct a disease activity scoring system for SLE patients by dividing the regression coefficients of the variables in the results of the multifactorial binary logistic regression analysis by the regression coefficient with the smallest absolute value (β-low complement=0.921) and rounding to the nearest whole number to arrive at the corresponding score for disease activity scoring in SLE patients.

We ultimately identified 25 variables as items for the new scoring model. Based on the scoring method mentioned earlier, the scores of these 25 items are as follows: Items with a score of 6: Vasculitis (X3); Items with a score of 5: Neuropsychiatric symptoms (X1) and Visual Impairment (X2); Items with a score of 4: Hematuria (X6), Proteinuria (X7); Items with a score of 3: Arthritis (X4), Myositis (X5), Hypothyroidism (X20), Hypocalcemia (X21), and Abnormal liver function (X23); Items with a score of 2: Pyuria (X8), Rash (X10), Mucosal Ulcers (X11), Pleurisy (X12), Pericarditis (X13), Elevated Anti-ds-DNA (X15), Leukopenia (X18), Pulmonary hypertension (X19), Lymphadenopathy (X22), and High-titer ANUA (X24); Items with a score of 1: Alopecia (X9), Hypocomplementemia (X14), Fever (X16), Thrombocytopenia (X17), and Decreased hemoglobin (X25).

The total SLE activity score is the sum of the risk scores corresponding to each of the aforementioned variables, ranging from 0 to 56 points. We calculated the activity score for all SLE patients according to this scoring method. Based on the derived activity scores and whether they corresponded to a PGA-defined moderate-to-severe SLE activity state, the Hosmer-Lemeshow goodness-of-fit test and ROC curve analysis were performed. The Hosmer-Lemeshow goodness-of-fit test yielded χ² = 3.109, P = 0.927 > 0.05, indicating good calibration of this scoring system. The ROC curve was plotted based on the predicted probability values; the AUC of the ROC curve was 0.969 (95% CI: 0.960–0.978, P < 0.001), indicating good discriminative ability of the scoring system. The optimal diagnostic cut-off value for the risk score was determined. The score cut-off value corresponding to the maximum Youden index of 0.806 was 10.5 points, rounding to integers 10 or 11 points. When setting 10 as the optimal cut-off value, the Youden index was 0.806(sensitivity 0.873, specificity 0.933). When setting 11 as the optimal cut-off value, the Youden index was 0.761 (sensitivity 0.798, specificity 0.963). A score of 10 points was ultimately selected as the optimal cut-off value.

### 3.5 Comparison of ROC curves between the newly developed predictive model and the SLEDAI-2K scoring system

Use ROC curves to evaluate the diagnostic performance of the newly developed scoring model and the SLEDAI-2K scoring system for patients with moderate to severe disease activity in SLE. According to the ROC curve results, both SLEDAI-2K and the newly developed scoring model demonstrate high diagnostic value for moderate to severe disease activity in SLE patients, with AUCs exceeding 0.9. Notably, the AUC of the new model (0.969 (95% CI: 0.960–0.978, P < 0.001)) is greater than that of SLEDAI-2K (0.919 (95% CI: 0.903–0.935, P < 0.001)). Therefore, it is recommended to adopt the new model for assessing disease activity in SLE patients, as it demonstrates superior performance compared to the SLEDAI-2K scoring model.

### 3.6 Correlation analysis of the newly developed SLE activity scoring model with the SLEDAI-2K and PGA scoring systems

**Figure 2:**
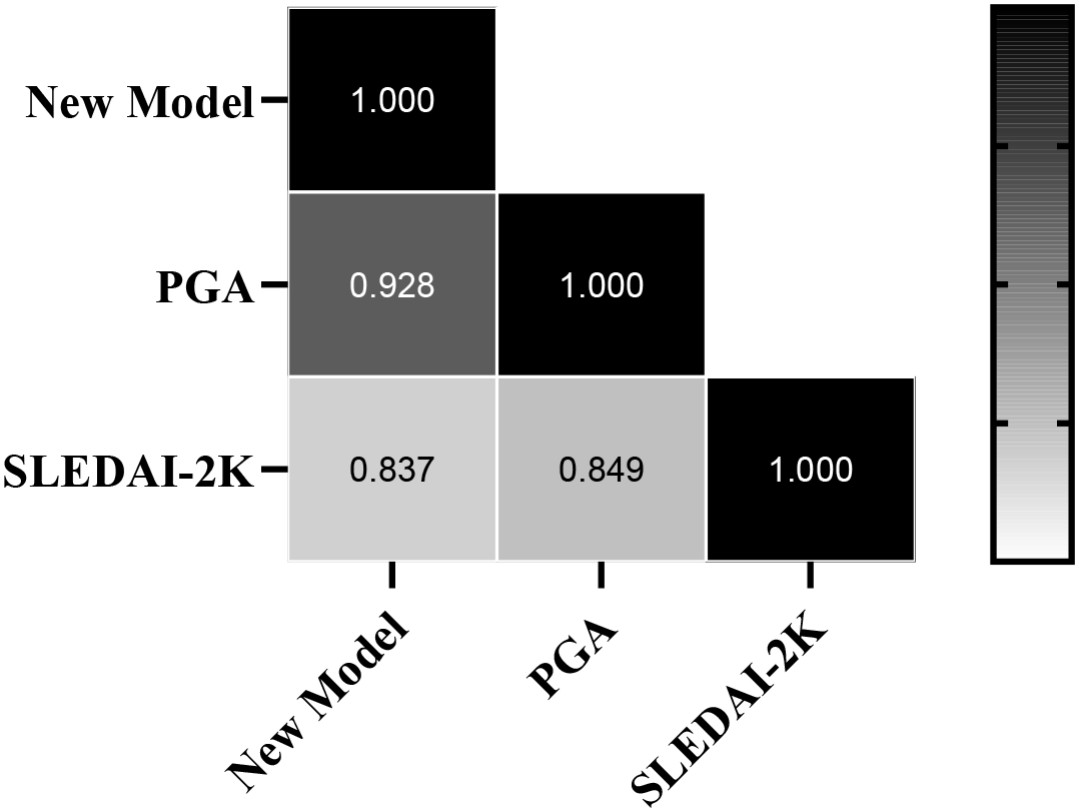
Correlation analysis of the new model with the SLEDAI-2K and PGA scoring systems.

Correlation analysis was performed between the scores obtained from the new scoring model and the SLEDAI-2K and PGA scores. Normality tests revealed that none of the three datasets followed a normal distribution; therefore, Spearman’s correlation coefficient was used to compare the correlations among the three scores. The scores from the new scoring model were highly correlated with the PGA score (r = 0.928, p < 0.001) and with the SLEDAI-2K score (r = 0.837, p < 0.001). Simultaneously, the SLEDAI-2K score was also highly correlated with the PGA score (r = 0.849, p < 0.001) (**Figure 2**).

### 3.7 External validation of the SLE activity scoring system

Clinical data from SLE patients hospitalized in our institution between June 2021 and December 2021 were collected, applying the same inclusion and exclusion criteria as before. A total of 323 SLE patients were successfully enrolled, comprising 153 patients (47.4%) with PGA-defined mild disease activity and 170 patients (52.6%) with PGA-defined moderate-to-severe disease activity. The activity score was calculated for each SLE patient according to the aforementioned scoring system. The Hosmer-Lemeshow goodness-of-fit test and ROC curve analysis were performed on the validation cohort scores. The Hosmer-Lemeshow goodness-of-fit test yielded χ² = 5.288, P = 0.726 > 0.05. The ROC curve was plotted based on the predicted probability values; the AUC of the ROC curve was 0.971 (95% CI: 0.958–0.985, P < 0.001), indicating that the diagnostic performance of this scoring system for moderate-to-severe SLE disease activity remained favorable in the validation cohort.

### 3.8 Discriminative ability of the new model for severe SLE activity

To further validate the model’s ability to discriminate severe disease activity (defined as PGA ≥ 2), we conducted an in-depth analysis. In the derivation cohort (n = 1,163), 409 patients (35.2%) met the criteria for severe activity. ROC curve analysis showed that the new model achieved a significantly higher AUC for discriminating severe activity (0.965; 95% CI: 0.955–0.974) compared to SLEDAI-2K (0.934; 95% CI: 0.921–0.947; P < 0.001). In the validation cohort (n = 323), 29 patients (9.0%) presented with severe activity. The new model maintained excellent discriminative ability, with an AUC of 0.986 (95% CI: 0.975–0.997), again outperforming SLEDAI-2K (AUC = 0.959; 95% CI: 0.934–0.983). These results demonstrate that the new model not only accurately identifies moderate-to-severe disease activity (PGA > 1) but also exhibits robust discriminative performance for severe activity (PGA ≥ 2), with consistent results across both strata.

### 3.9 Subgroup analysis: performance of the new model in specific severe SLE manifestations

To further evaluate the discriminative ability of the new model in specific severe SLE manifestations, subgroup analyses were performed in patients with active lupus nephritis and those with active neuropsychiatric SLE (NPSLE). In the derivation cohort (n=1163), a total of 444 patients presented with active lupus nephritis. Within this subgroup, using PGA-defined moderate-to-severe disease activity (PGA >1) as the gold standard, the new model demonstrated excellent discriminative ability with an AUC of 0.982 (95% CI: 0.972–0.992), which was significantly higher than that of SLEDAI-2K (AUC = 0.937, 95% CI: 0.914–0.960; P < 0.001). In the NPSLE subgroup, 80 patients were identified as having active neuropsychiatric involvement. Despite the limited sample size, the new model maintained robust discriminative performance with an AUC of 0.981 (95% CI: 0.947–1.000), comparable to SLEDAI-2K (AUC = 0.977, 95% CI: 0.947–1.000). These findings indicate that the new model not only performs well in the overall SLE population but also exhibits excellent discriminative ability in specific severe manifestations such as active lupus nephritis and active NPSLE, with superior or comparable performance to SLEDAI-2K across these clinically important subgroups.

## 4 Discussion

This study constructed a new SLE disease activity scoring model based on a multicenter cross-sectional design and binary logistic regression analysis. Focusing on multiple clinical manifestations influencing SLE disease activity, it proposes a more precise scoring system.

First, by analyzing data from 1163 SLE patients, this study identified 25 clinical manifestations as important independent risk factors influencing SLE disease activity. Ultimately, an SLE disease activity assessment system encompassing 25 influencing factors was established. Compared to SLEDAI-2K, this study is the first to jointly incorporate manifestations of involvement from the circulatory, endocrine, hematologic, and digestive systems—specifically pulmonary hypertension, hypothyroidism, hypocalcemia, lymphadenopathy, abnormal liver function, and decreased hemoglobin—into the SLE activity scoring system. This expansion more comprehensively reflects the pathological characteristics of multi-system involvement in SLE.

In this study, PAH that is new or significantly progressed within 10 days was incorporated into the assessment of SLE activity, with this definition specifically referring to arterial pulmonary hypertension (12). Evidence suggests a close association between PAH and SLE disease activity. The prevalence of PAH is significantly higher in patients with SLE compared to the general population (13); furthermore, inflammatory signaling pathways (e.g., interferons, TNFα) play a central role in the pathogenesis of SLE-PAH (14). Moreover, clinical studies have confirmed that disease activity markers such as SLEDAI score and anti-dsDNA antibodies are independent predictors of PAH in SLE patients (15), and combined immunosuppressive therapy with targeted PAH treatment significantly improves patient outcomes (16). As one of the leading causes of death in SLE patients, early identification and intervention for PAH are crucial for improving long-term prognosis. Incorporating PAH into the new scoring model addresses the neglect of circulatory system involvement by SLEDAI-2K and aligns closely with the holistic principle of the PGA.

Thyroid hormones are essential for multisystem metabolism, and their abnormalities can exacerbate organ damage in SLE (17, 18). The inclusion of hypothyroidism is supported by multiple lines of evidence: the prevalence of hypothyroidism is significantly increased in SLE patients and independently associated with higher SLEDAI scores, renal involvement, and anti-dsDNA positivity (19, 20). Thyroid hormone levels also fluctuate with disease activity—in patients with non-thyroidal illness syndrome (NTIS), FT3 levels negatively correlate with SLEDAI and proteinuria (21); elevated inflammatory markers are independent risk factors for NTIS (22), suggesting that systemic inflammation may influence thyroid function by suppressing the hypothalamic-pituitary-thyroid axis. Furthermore, a bidirectional causal relationship and shared genetic susceptibility may exist between SLE and hypothyroidism (23). In this study, hypothyroidism was identified as an independent risk factor; incorporating it into the new model not only compensates for the limitations of SLEDAI-2K in assessing endocrine involvement and enhances content validity, but also better aligns with the holistic principle of PGA.

Clinical observations indicate that digestive system involvement occurs in approximately 17.7%-50% of SLE cases, with about 10%-13.1% presenting digestive symptoms as the initial manifestation (24). The liver, as a significant immune regulatory organ, is susceptible to immune-mediated damage in SLE. Current research has revealed potential links between SLE and the liver from the perspectives of the microbiome and immune activation, while the systemic inflammatory state in SLE patients can affect the liver via the circulatory system (25). These findings suggest that impaired liver function may reflect SLE disease activity. Given the low specificity of liver injury-related symptoms (e.g., nausea, abdominal distension, fatigue), this study utilized routinely measured serum enzyme markers—aspartate aminotransferase (AST), alanine aminotransferase (ALT), and gamma-glutamyl transferase (GGT)—as objective quantifiable evidence of liver function impairment (26). The results confirm that abnormal elevation of these enzymes is an independent risk factor for moderate-to-severe SLE activity. Therefore, by incorporating these readily available laboratory indicators, the model successfully and conveniently integrates digestive system involvement into the comprehensive assessment of SLE activity.

Lymphadenopathy, hypocalcemia, and decreased hemoglobin are common manifestations in SLE; however, due to their low specificity, they are often overlooked by traditional assessment tools (27, 28). Through rigorous statistical methods, this study successfully incorporated these indicators into the scoring system. Taking hypocalcemia as an example: serum calcium levels have been shown to fluctuate synchronously with SLE activity, serving not only as an independent marker of disease activity but also as an indicator where recovery often accompanies treatment response (28); SLE patients with hypocalcemia exhibit increased numbers of peripheral blood CD8^+^ T cells with an activated phenotype, along with elevated Th1-type cytokines, suggesting enhanced cellular immunity (29); furthermore, vitamin D deficiency, commonly present during active disease, can also lead to hypocalcemia and is negatively correlated with SLEDAI (30). Consistent with previous studies, incorporating systemic manifestations such as lymphadenopathy into the model has also been shown to improve its correlation with the PGA (31).

Autoantibodies are key indicators in the assessment of SLE (32). After analyzing more than ten autoantibodies, this study identified high-titer ANUA as an independent risk factor for moderate-to-severe SLE activity. The inclusion of ANUA is well-supported: its production stems from the core pathology of SLE—nucleosomes released by apoptotic cells drive autoantibody generation, and the deposited immune complexes lead to tissue damage (33); ANUA levels are closely associated with disease activity, significantly elevated in active SLE and lupus nephritis, and positively correlated with SLEDAI (33, 34); longitudinal studies have confirmed that ANUA fluctuates with disease course, decreases after treatment, and its changes are strongly correlated with changes in SLEDAI (r = 0.629) (34); meta-analyses suggest that ANUA may have superior diagnostic and prognostic value compared to anti-dsDNA antibodies (35). Incorporating high-titer ANUA into the model enhances the ability to assess overall SLE activity, addresses the limitations of traditional tools in dynamic immunological monitoring, and provides a novel clinical indicator.

In model validation, the AUCs of the ROC curves were close to 1.0 in both the derivation and validation cohorts. This result demonstrates that the new scoring model can sensitively identify moderate-to-severe activity states in SLE patients, showing higher sensitivity particularly when compared with SLEDAI-2K. This is crucial for clinicians to identify patients with moderate-to-severe SLE activity early in clinical practice and promptly adjust treatment strategies. Furthermore, this study also validated the applicability and consistency of the new model across different cohorts. In both the derivation and validation cohorts, the H-L test results indicated good model calibration, suggesting high reliability across different patient populations. This lays the foundation for the practical clinical application and dissemination of this scoring model.

Although this study provides a new model for SLE disease activity assessment, certain limitations exist. First, the model was developed and validated only in a Han Chinese population from the Shandong region, without external validation in other racial or geographic cohorts. Multicenter international collaboration is needed in the future to conduct cross-ethnic validation. Second, the cross-sectional study design limits the assessment of the reversibility of clinical manifestations, and the relatively small sample size in certain subgroups may affect the stability of subgroup analyses. Large-scale longitudinal studies with more diverse cohorts are warranted in the future to further validate our findings.

In conclusion, this study successfully constructed and validated a new SLE disease activity scoring model. By integrating clinical manifestations of multiple system involvement and key autoantibody indicators, the model significantly enhances sensitivity in identifying patients with moderate-to-severe activity while maintaining high specificity. Its favorable ease of use and validation performance endow it with significant clinical value and broad prospects for dissemination in optimizing the management and treatment decision-making for SLE patients.

## Data Availability

The raw data supporting the conclusions of this article will be made available by the authors, without undue reservation.

## Glossary

SLE: Systemic Lupus Erythematosus
SLEDAI-2K: SLE Disease Activity Index 2000
PGA: Physician Global Assessment
ANUA: anti-nucleosome antibodies
ACR: American College of Rheumatology
ESR: Erythrocyte Sedimentation Rate
hs-CRP: high-sensitivity C-reactive protein
ROC: Receiver Operating Characteristic
AHA: anti-histone antibodies
ALB: albumin
ANA: antinuclear antibodies
AnuA: anti-nucleosome antibodies
anti-dsDNA: anti-double-stranded DNA antibodies
anti-rRNP: anti-ribosomal P protein antibodies
anti-Sm: anti-Smith antibodies
anti-SSA/anti-SSB: anti-Sjögren’s-syndrome-related antigen A/B antibodies
anti-U1RNP: anti-U1 ribonucleoprotein antibodies
AUC: area under the curve (of ROC)
BILAG-2004: British Isles Lupus Assessment Group Index-2004
BUN: blood urea nitrogen
CI: confidence interval
CRP: C-reactive protein
CTD-PAH: connective tissue disease–associated pulmonary arterial hypertension
ECLAM: European Consensus Lupus Activity Measurement
ESR: erythrocyte sedimentation rate
GGT: gamma-glutamyl transferase
IgA/IgG/IgM: immunoglobulin A/G/M
IQR: inter-quartile range
OR: odds ratio
SLAM: Systemic Lupus Activity Measure
SRI: SLE Responder Index
AST: Aspartate Aminotransferase
ALT: Alanine Aminotransferase
GGT: Gamma-glutamyl transferase.
